# Basal Blood Morphology, Serum Biochemistry, and the Liver and Muscle Structure of Weaned Wistar Rats Prenatally Exposed to Fumonisins

**DOI:** 10.3390/ani12182353

**Published:** 2022-09-08

**Authors:** Ewa Tomaszewska, Halyna Rudyk, Dorota Wojtysiak, Janine Donaldson, Siemowit Muszyński, Marcin B. Arciszewski, Nataliia Lisova, Oksana Brezvyn, Iwona Puzio, Beata Abramowicz, Marta Pawłowska-Olszewska, Ihor Kotsyumbas, Piotr Dobrowolski

**Affiliations:** 1 Department of Animal Physiology, Faculty of Veterinary Medicine, University of Life Sciences in Lublin, Akademicka 12, 20-950 Lublin, Poland; 2 State Scientific Research Control Institute of Veterinary Medicinal Products and Feed Additives, Donetska 11, 79000 Lviv, Ukraine; 3 Department of Animal Genetics, Breeding and Ethology, Faculty of Animal Sciences, University of Agriculture in Kraków, Mickiewicza 24/28, 30-059 Cracow, Poland; 4 School of Physiology, Faculty of Health Sciences, University of the Witwatersrand, 7 York Road, Parktown, Johannesburg 2193, South Africa; 5 Department of Biophysics, Faculty of Environmental Biology, University of Life Sciences in Lublin, Akademicka 13, 20-950 Lublin, Poland; 6 Department of Animal Anatomy and Histology, University of Life Sciences in Lublin, Akademicka 12, 20-950 Lublin, Poland; 7 Department and Clinic of Animal Internal Diseases, Faculty of Veterinary Medicine, University of Life Sciences in Lublin, Głęboka 30, 20-612 Lublin, Poland; 8 Department of Functional Anatomy and Cytobiology, Faculty of Biology and Biotechnology, Maria Curie-Sklodowska University, Akademicka 19, 20-033 Lublin, Poland

**Keywords:** fumonisins, prenatal exposure, liver, heart, striated muscles, developmental changes, structural changes, organs inflammation, metabolic changes

## Abstract

**Simple Summary:**

Cereal, which is the main ingredient of animal feed, is often contaminated with mold, which produces heat-resistant, carcinogenic, and harmful metabolites/toxins called fumonisins. Feed contamination with fumonisins is a worldwide problem; however, the dietary intake of fumonisins is difficult to estimate because their concentrations in many products are unknown. The effects of consuming fumonisin-contaminated feed on animal health are not fully known, and the economic losses that are related to health care or animal husbandry are difficult to calculate as fumonisins are found commonly in foods, including those that are intended for infants or pregnant dams. The involuntary intake of moldy feed leads to a serious health risk with long-term effects. The research on prenatal exposure to fumonisins is limited. Previous studies have shown that prenatal fumonisins exposure causes abnormalities in the bone and enteric nervous system development. Therefore, it is very important to study the effects of prenatal exposure to fumonisins on the general development of offspring at different periods of life, including weaning.

**Abstract:**

Cereals are often contaminated with fumonisins, which are the toxic byproducts of mold. The aim of the study was to determine the effect of maternal exposure to fumonisins on the development and the liver function of the offspring at weaning. Two doses of fumonisins (60 and 90 mg/kg b.w.) were tested. The changes in the basal blood morphology, the biochemical parameters, the absolute and relative weights of the vital organs, and the changes in the cardiac and biceps brachii muscle histology were studied. The liver damage was assessed by evaluating the liver morphology and the common clinical liver panel. Maternal fumonisin intoxication caused a decrease in the body weight at birth and an increase in the heart, liver, kidney, lungs, ovaries, and testes weights. The cytokines and hormones, as well as the red blood cell counts and hemoglobin levels, were elevated in a dose-dependent manner following the exposure to fumonisins. Maternal exposure caused degenerative morphological and structural changes in the liver, as well as inflammation in the striated muscles, such as the heart and biceps brachii, and disproportionate development of the rat offspring in a dose-dependent manner. Moreover, FB exposure resulted in the disproportional development of the rat offspring in a dose-dependent manner, which was probably caused by the bodily hormonal dysregulation. Prenatal fumonisin exposure can be a pathological precursor for serious diseases, such as obesity and diabetes, later in life.

## 1. Introduction

Cereal, which is the main component of animal feed, is often contaminated with mold, among which is the common *Fusarium* strain, which produces heat-resistant metabolites/toxins called fumonisins (A, B, C, and P). Type B fumonisins (FB1 and FB2) are common and are very toxic. Type FB2 is a cytotoxic analog of FB1, and it occurs naturally in a ratio of FB2 to FB1 at about 1:3 [1]. The type B fumonisins act as inhibitors of sphingosine N-acyltransferase. FB2 also inhibits the protein serine/threonine phosphatase [2]. The basic mechanism by which they show their toxicity is through their interference with sphingolipid metabolism [3,4]. The International Agency for Research on Cancer (IARC) has designated FB1 in group 2B, meaning that it is “possibly carcinogenic to humans” [5]. FB are also hazardous to animals due to their causative factors of neurotoxicity, kidney toxicity, immunosuppression, and hepatocarcinogenesis [6,7]. The clinical signs of FB poisoning depend on the dose and the route of exposure, as well as the sex, the age, and the species of the animal. Some animals display non-species-specific symptoms (rodents, sheep, pigs, horses, and poultry) or symptoms relating strictly to certain target organs (pigs—the lungs and the esophagus, horses—the brain), while others, such as ruminants, whose rumen microbiota degrade mycotoxins, are resistant to the effects of FB [8,9,10,11,12,13,14]. The contamination of feed with FB is a worldwide problem, and for that reason the total amount of FB that is acceptable in feedstuff is regulated by the European Food Safety Authority (EFSA) and EU legislation (Directive 2003/100/EC and Recommendation 2006/576/EC) and by the FDA in the USA [15,16,17]. However, the dietary intake of FB is difficult to assess since FB are also produced by *Aspergillus niger* [5] and their concentration in many products is unknown due to either improper cereal storage or infrastructure contamination. The primary cause of FB contamination of livestock feed is the lack of hygiene, relating not only to the storage and the feed preparation, but also to the farmlands, the transport procedures, and the outdoor and indoor environments, all of which are often microbiologically contaminated. All of these stages these are indispensable parts of the animal production process. The influence of the intake of feed that is contaminated with FB on animal health is not fully known. Moreover, the economic losses in the provision of health care or animal husbandry are difficult to calculate because, as previously mentioned, FB is found in moldy food that is intended for animals, including infants or pregnant mothers. The involuntary consumption of moldy feed is a major health risk, especially given the role that optimal nutrition plays in prenatal development and its long-term effects later in life [12,18,19,20]. Research concerning prenatal exposure to FB is limited. Previous studies have shown that exposure to FB results in disturbances in the bone and enteric nervous system development [18,19,20,21]. Given the direct deleterious effects of FB on many of the body systems in non-human vertebrates and the indirect effects of maternal FB exposure during pregnancy on the newborn offspring, it seems reasonable to study the effects of FB exposure during pregnancy on the overall development of the offspring at weaning.

Therefore, the main goal of the current study was to assess the effects of maternal FB intoxication on the general health status of weaned rats. The study has examined the changes in the common clinical liver panel parameters (aspartate transaminase, alanine transaminase, bilirubin, alkaline phosphatase, and gamma-glutamyltranspeptidase) and the liver structure, as well as the basal blood morphology and other blood serum biochemical parameters. The changes in the histology of the striated muscles (the heart tissue and the skeletal muscles) were also assessed. All of these analyses should provide some fundamental knowledge regarding the outcomes of FB exposure during pregnancy on the postnatal development of and the degree of liver cell damage in weaned offspring.

## 2. Materials and Methods

The experiment was performed in accordance with the EU Directive 2010/63/EU, under the license of the State Scientific Research Control Institute of Veterinary Medicinal Products and Feed Additives in Lviv, Ukraine.

### 2.1. Fumonisins Preparation and Quantification 

The FBs were produced in vitro on a maize grain medium with the use of *F. moniliforme*, as previously described [12]. Briefly, autoclaved, coarsely cracked grains were inoculated with *F. moniliforme* cultures and were cultivated for 4 weeks at 24 °C. The contaminated maize was then autoclaved, dried, ground, and analyzed for FB1 and FB2 using liquid chromatography, which showed the typical 3:1 ratio of FB1 and FB2 (73% to 27%). Next, the FBs were extracted from the ground grains with an ethanol solution, were quantified using an ELISA (#R3401, Ridascreen Fumonisin, R-Biopharm AG, Darmstadt, Germany), and were concentrated to 100 mg/mL FB1 + FB2 stock solution. During the experiment, the FB extract stock was diluted in 0.9% saline solution to yield the necessary concentration in 0.5 mL on the basis of the daily measurements of individual rat weight.

### 2.2. Animals and Experimental Design

Pregnant Wistar rat dams (n = 18; 5-weeks-old) were housed individually in polypropylene cages (the dimensions of 380 × 200 × 590 mm). During a one-week acclimatization period under laboratory conditions, the dams were kept at a temperature of 21 ± 3 °C and a humidity of 55 ± 5%, with a 12 h/12 h day/night cycle, and were fed a standard laboratory rodents’ diet ad libitum, with free access to water. After the acclimatization period, the rats were randomly allocated to either a control group, which was not treated with FB (C group; n = 6) or to one of the two other groups that were intoxicated with FB at a dose of 60 mg FB/kg b.w. or 90 mg FB/kg b.w. (n = 6 in each group). A standard laboratory rodents’ diet was offered to the pregnant dams, and the animals were fed ad libitum. The fumonisins were given by daily intragastric administration in 0.5 mL of 0.9% saline solution, from the 7th day of pregnancy up to parturition [12,13]. The control animals received saline solution in the corresponding amount and manner. The 90 mg FB/kg b.w. dose was equal to 1/10 of the established LD_50_ dose [12,18] and was sufficient to induce subclinical intoxication in adolescent rats [12,18]; while the 60 mg dose exceeded the dose required to trigger embryonic neural tube defects when FB were given before the 7th day of pregnancy, was equal to 1/15 of the established LD_50_ value, and did not induce subclinical or clinical symptoms in adolescent rats [12]. Lower FB doses have been studied extensively (0.1, 0.5, 1.9, 3.8, 6.3, 10, 12, 15, 18, 25, 30, 45, and 50 mg/kg b.w., given in different gestational days) [22]. The higher dose was not used because it results in the presence of clinical signs and for this reason permission from the ethical committee was not received. A trained veterinarian did not note any changes in pregnant dam behavior or basal health state. Following parturition, all newborns were weighed and kept with their mothers, without translocation between the litters. All newborns were divided according to their mothers as follows: the 0 FB group were considered the controls; the 60 FB group were prenatally exposed to FB at a dose of 60 mg/kg b.w.; and the 90 FB were prenatally exposed to FB at a dose of 90 mg/kg b.w. At weaning, at the age of 28 days, four offspring from each mother (two males and two females) (n = 12 males and n = 12 females in total) were weighed and euthanized by CO_2_ inhalation.

### 2.3. Organ Weights

After the euthanasia, immediately after the collection of blood samples, the animals were dissected and various organs, including the liver, lungs, heart, testes, ovaries, and kidneys, were removed and weighed individually. The relative organ weight was determined. It is accepted practice in presenting organ weight data to express the results relative to the animal’s body weight.

### 2.4. Blood Measurements

The whole blood was collected by intracardiac puncture into tripotassium salt of ethylenediaminetetraacetic acid (K_3_EDTA) coated tubes (BD Vacutainer Systems, Plymouth, UK) for hematology and into vacutainer tubes with cloth activator for blood serum determinations (1300× *g* for 10 min at 18 °C). The collected serum was aliquoted into polypropylene tubes and stored at −86 °C until the assays were performed.

The number of white blood cells (WBC), lymphocytes, monocytes, neutrophils, red blood cells (RBC), and platelets (PLT), as well as the hemoglobin concentration (Hb), hematocrit (HCT), mean corpuscular volume (MCV), mean corpuscular hemoglobin (MCH), and mean corpuscular hemoglobin concentration (MCHC) were determined using an automatic hematology analyzer (Advia 2120, Siemens Healthcare, Erlangen, Germany).

The serum was analyzed for the following common markers of liver damage: alanine transaminase (ALT), aspartate transaminase (AST), gamma-glutamyl transpeptidase (GGT), and alkaline phosphatase (ALP); and the following other serum parameters: creatine kinase (CK), total bilirubin (TBIL), total cholesterol (TCHOL), glucose, total protein (TP), calcium (Ca), phosphorus (P), magnesium (Mg), and lactate dehydrogenase (LDH), using an automatic analyzer (Mindray BS-120, Bio-Medical Electronics, Shenzhen, China) and ready-to-use commercial tests (Alfa Diagnostics, Warsaw, Poland). All analyses performed were verified with the usage of multiparametric control serum (Alfa Diagnostics, Warsaw, Poland).

The serum concentrations of growth hormone (GH), interleukin 1β (IL-1β), and interleukin 6 (IL-6) were determined using commercial, rat-specific, enzyme-linked immunosorbent assay (ELISA) kits from BT-Lab (Korain Biotech, Shanghai, China). The serum insulin (INS) was determined using a commercial, rat-specific, ELISA kit from Qayee-bio (QY-E11704; Qayee-bio, Shanghai, China). All procedures were performed in accordance with the manufacturers’ protocols. The analysis of the samples was performed in duplicates, using a microplate spectrophotometer (Benchmark Plus, Bio-Rad Laboratories, Inc., Hercules, CA, USA). To calculate particular results, the individual standard curves that were created in individual tests were used.

### 2.5. Tissue Sampling and Histological Analysis

The samples from the center of the right dorsal lobe of the liver were snap frozen in liquid nitrogen and stored at −80 °C until subsequent analyses. The heart (left ventricle) and a section from the biceps brachii muscles were fixed in 4% buffered formaldehyde (pH 7.0) for 24 h. The formaldehyde-fixed samples were fixed in paraffin according to a routine procedure. The samples were cut with a microtome, depending on the target, into 6-μm (Leica RM2146 microtome, Nußloch, Germany) or 4-µm (Microm HM 360, Microm, Walldorf, Germany) sections and were stained with Goldner trichrome, and hematoxylin & eosin (H&E) (Sigma-Aldrich, Darmstadt, Germany) for morphological examination using light microscopes (CX43 and BX63, Olympus, Tokyo, Japan). The micromorphology of the liver was examined under microscopic observation and the images were collected with the use of graphical analysis software, CellSens Olympus Version 1.5 (OLYMPUS, Tokyo, Japan). A square 9-field grid was used to take consecutive images of each section, and images were taken from the four corner fields and the center field. The microscopic observations identified and evaluated the normal structure of the liver, including the portal triad and terminal hepatic veins, for assessing the architecture of the lobules. Mature fibrous tissue, portal tract stroma, and immature fibrous tissue, as well as lobular architecture and small hepatocytes (as characteristic of regeneration), were also assessed. The microscopic observations also allowed us to identify ballooning degeneration. In addition to the histopathological examination, the total hepatocyte number, the number of binucleated hepatocytes, and the non-hepatocyte cell number were determined on three separate tissue sections, on at least ten different areas of each section, using image analysis software ImageJ 1.53 (National Institute of Health USA, http://rsb.info.nih.gov/ij/index.html; accessed on 24 July 2022). The measurements were then averaged and were expressed as the mean value of the calculated parameters for each rat [23,24]. The histological sections of the left ventricle myocardium and the bicep muscle were analyzed under microscopic observation (Nikon E600, Tokyo, Japan) and inflammatory cell infiltration was assessed semi-quantitatively (0 = lack, 1 = low, 2 = moderate, 3 = high, and 4 = severe). All histological evaluations were performed independently by three histologists with more than 20 years of experience in the field (D.W., E.T., and P.D.).

### 2.6. TUNEL (Terminal Deoxynucleotidyl Transferase Biotin–dUTP Nick end Labeling) Assay

The apoptotic nuclei were detected using the ApopTag Plus Peroxidase In Situ Apoptosis Detection Kit (Chemicon International, Melbourne, Australia) following the manufacturer’s protocol. Briefly, after deparaffinization and rehydration, slides were pretreated with proteinase K solution (10 μg/mL, Promega Corporation, Madison, WI, USA) for 15 min at room temperature (RT), followed by incubation at RT for 10 min in 3% H_2_O_2_ in methanol to quench endogenous peroxidase activity. The slides were then incubated in equilibration buffer for 10 min at RT in a humid chamber, after which the enzyme TdT (terminal deoxynucleotidyl transferase) was added at working strength and they were incubated for 1 h in a humid chamber at 37 °C. The slides were then washed at RT with wash buffer for 10 min, followed by washing with PBS (phosphate-buffered saline) for 5 min. After the excess fluid was drawn off, anti-digoxigenin conjugate was applied directly to the slides and they were incubated for 30 min at RT in a humidified chamber. After washing in PBS, apoptotic cells were visualized by adding DAB (3,3′-diaminobenzidine) solution. A negative control was performed without active TdT enzyme to control for non-specific incorporation of nucleotides or for non-specific binding of enzyme conjugate. All of the sections were counterstained with Mayer’s hematoxylin, dehydrated through an increased series of ethanol, and were mounted under glass with DPX (dibutylphthalate polystyrene xylene, Sigma Chemical Co, St. Louis, MO, USA). The TUNEL-positive nuclei were counted on 10 random areas of each liver tissue and the results were expressed as the percentage of apoptotic cells per 100 randomly counted liver cells. The sections were analyzed under a Zeiss Axio Imager A.2 light microscope (Carl Zeiss AG, Jena, Germany) and AxioVision Digital Image Processing System version 4.8.2 (Carl Zeiss AG, Jena, Germany).

### 2.7. Beclin-1 Immunostaining

The liver tissues for immunohistochemical analysis were frozen in liquid nitrogen and stored at −80 °C for later analysis. The samples were mounted on a cryostat holder using tissue-freezing medium (Tissue-Tek; Sakura Finetek Europe, Alphen aan den Rijn, The Netherlands). The frozen tissue was cut into 10-μm-thick slices at −20 °C in a cryostat (Slee MEV, Mainz, Germany). Beclin-1 activity was determined on frozen sections that were fixed with 4% formaldehyde, as paraformaldehyde (PFA), in 0.1M phosphate buffer (PB) (pH 7.4), and then were incubated for 30 min in 5% normal goat serum (NGS). They were then incubated overnight at 4 °C with anti-Beclin-1 monoclonal antibody (sc-48341, Santa Cruz Biotechnology, Dallas, TX, USA; dilution 1:100).The slides with control for primary antibody were incubated with PBS instead of primary antibody. After several washes in 0.01M sodium phosphate buffer (PBS) containing 0.5% Triton-X, sections were incubated overnight at 4 °C with goat anti-mouse secondary antibodies conjugated to Alexa Fluor 488 (A11001, Thermo Fisher Scientific, Waltham, MA, USA; dilution 1:1000). After final washing, the slides were mounted with Vectashield mounting medium (Vector Labs, Burlingame, CA, USA) with DAPI and were examined with a Zeiss Axio Imager A.2 (Carl Zeiss AG, Jena, Germany) fluorescence microscope and AxioVision Digital Image Processing System version 4.8.2 (Carl Zeiss AG, Jena, Germany).

### 2.8. Statistical Analysis

The results are expressed as means ± SEM. At the beginning, the effect of the mother was examined by nested ANOVA, where the offspring were nested into the relevant mothers. Since there was no significant effect of the mothers detected, the rest of the analysis was carried out using a two-way ANOVA, followed by Tukey’s multiple comparison post-test. Planed comparisons were used to check the linear and quadratic effects. The Shapiro–Wilk test and the Brown–Forsythe test were performed to check the assumptions of normal distribution of the data and the equality of the variance, respectively. All statistical analyses were carried out using data analysis software system STATISTICA (ver. 12, StatSoft, Inc., Tulsa, OK, USA). In cases where data lacked normal distribution and/or equality of variance, the non-parametric Kruskal–Wallis and median tests were applied. The following statistical model was used to analyze the selected parameters:

x_ij_ = µ + α_i_ + β_j_ + (αβ)_ij_ + ε_ijk_ (1)

where x_ij_—observation (measured parameter), i—the level of the first factor (0 mg FB/kg b.w., 60 mg FB/kg b.w., and 90 mg FB/kg b.w.), j—level of the second factor, sex (female or male), k—number of measurements, µ—constant, general mean, α_i_—main effect of the first factor, β_j_—main effect of the second factor, (αβ)_ij_—interaction effect of the main factors, and ε_ijk_—random error. *p*-values less than 0.05 were considered statistically significant.

## 3. Results

### 3.1. Body Mass and Absolute and Relative Liver Mass of Weaned Rats

In the female offspring, the FB exposure caused a large decrease in their body weight at birth, as was confirmed by a significant linear effect. In contrast, the lower dose of FB mainly caused an increase in the weaning weight in the female offspring, as was confirmed by a significant quadratic effect (Table 1).

The maternal FB intoxication at a dose of 60 mg/kg b.w. resulted in an increase in the relative ovaries weight compared to that observed in the control group and the higher FB dose group, with all evidenced by significant quadratic and linear effects. The heart and the liver relative weights were decreased after FB intoxication, with the linear effect being statistically significant. There were no significant differences in the kidney or the lungs relative weights, although a linear decrease in the relative kidney weight was observed (Table 1).

As with the females, the FB exposure also caused a large decrease in the body weight at birth in the male offspring, as was confirmed by a significant linear effect. The low dose of FB, however, caused an increase in the post-weaning body weight in the male offspring, as was confirmed by a significant quadratic effect (Table 1).

The maternal FB intoxication at a dose of 60 mg/kg b.w. resulted in an increase in the relative testis weight compared to that of the control group and the high FB dose group, as was evidenced by significant quadratic and linear (except for the liver) effects. The low FB dose increased the relative liver weight compared to that observed in the high FB dose group, although both of the groups were not significantly different from the control group, with a quadratic effect being evident. The maternal exposure to both of the FB doses substantially decreased the relative lungs weight in the male offspring. No effect of maternal FB intoxication was observed in the relative heart, kidney, or lungs weights.

Weight loss at birth was gender-dependent, as the FB intoxication caused greater weight loss in the males (*p* = 0.005).

### 3.2. Biochemistry

The basal biochemical parameters, including the cytokines and the hormones, are presented in Table 2.

The insulin concentration in the female weaned rats was highest in the group that was intoxicated with FB at a dose of 90 mg/kg b.w. (linear and quadratic, *p* < 0.001 and *p* < 0.001, respectively). The IL-1 concentration was significantly higher in the group that was intoxicated with FB at a dose of 90 mg/kg b.w. compared to that of the 0 FB group (linear, *p* = 0.009); while the IL-6 concentration was significantly higher in the group that was intoxicated with FB at a dose of 60 mg/kg b.w. compared to that of the 90 mg/kg b.w. FB group (quadratic, *p* = 0.021). A significant linear decrease in the GH concentration was observed after the prenatal FB exposure (*p* < 0.001), regardless of the dose. The serum glucose concentration was significantly higher in the offspring from the 60 mg/kg b.w. FB group compared to that of the other groups (linear and quadratic, *p* = 0.007 and *p* < 0.001, respectively). The concentration of the total protein was higher in both of the FB-intoxicated groups compared to the 0 FB group (linear and quadratic, *p* < 0.001 and *p* = 0.003, respectively). A significant linear increase in the ALAT activity was observed in the female offspring after the prenatal FB exposure, regardless of the dose (linear and quadratic, *p* < 0.001 and *p* = 0.029, respectively). A significant linear decrease in the ALP was also noted (*p* < 0.001). The activity of the ALP and the LDH decreased after the prenatal FB exposure (ALP—linear, *p* < 0.001) and the LDH level was increased by both of the FB doses; however, no linear or quadratic effects were noted. The activity of the CK was increased in both of the FB-intoxicated groups, with the highest activity being noted in the 60 mg/kg b.w. FB group (linear and quadratic, for both *p* < 0.001). A significant quadratic increase in the GGT was observed, with higher GGT activity being observed in the FB group that was intoxicated with a dose of 60 mg/kg b.w. compared to the other groups (*p* < 0.001). No significant differences in the Ca concentration were observed between the groups. The concentration of the P and the Mg decreased after the prenatal FB exposure (linear and quadratic, *p* = 0.003 and *p* = 0.013 for P; and *p* = 0.003 and *p* = 0.001 for Mg, respectively).

With regards to the male offspring, a significantly higher insulin concentration was observed in the 90 mg/kg b.w. FB group compared to that of the other groups (linear and quadratic, for both *p* < 0.001). The IL-1 was significantly higher in the 60 mg/kg b.w. FB group compared to the 90 mg/kg b.w. FB group (quadratic, *p* = 0.003) and the concentration of the IL-6 was significantly lower in the 60 mg/kg b.w. FB group compared to the 0 FB group (quadratic, *p* = 0.007). A significant linear decrease in the GH concentration was observed after the prenatal FB exposure (*p* < 0.001). The highest serum concentration of glucose was observed in the male offspring in the 60 mg/kg b.w. FB group, while the lowest glucose concentration was observed in the offspring in the 90 mg/kg b.w. FB group (linear and quadratic, *p* = 0.025 and *p* < 0.001). Since the lower dose of FB tended to increase the ASPT activity, and the higher dose tended to decrease the ASPT activity, a quadratic effect was noted (*p* = 0.043). The activity of the ALAT increased after the prenatal FB intoxication (linear and quadratic, *p* = 0.001 and *p* < 0.001) and the activity of the ALP was higher in the 60 mg/kg b.w. FB group compared to that in the 90 mg/kg b.w. FB group (linear and quadratic, *p* < 0.001 and *p* < 0.001). The activity of the LDH was significantly higher in the 60 mg/kg b.w. FB group compared to that in the 0 FB group and 90 mg/kg b.w. FB group (quadratic, *p* < 0.001). The activity of the CK increased after FB exposure (linear and quadratic, *p* < 0.001 and *p* < 0.001) and the activity of the GGT was lower in the 60 mg/kg b.w. FB group compared to that of the 0 FB group and the 90 mg/kg b.w. FB group (quadratic, *p* < 0.001). The prenatal exposure to FB at a dose of 60 mg/kg b.w. significantly increased the serum Ca concentrations; while exposure to 90 mg/kg b.w. FB significantly decreased the serum Ca concentrations compared to the 0 FB group (*p* = 0.013 and *p* < 0.001). No other changes in the serum parameters were observed.

The changes that were due to maternal FB intoxication were gender-dependent in more than half of the biochemical variables that were assessed, such as the IL-1 (*p* = 0.009), the IL-6 (*p* = 0.001), the glucose (*p* = 0.004), the total protein (*p* < 0.001), the ASPT (*p* < 0.001), the ALP (*p* = 0.001), the CK (*p* = 0.038), the Ca (*p* < 0.001), P (*p* = 0.011), the Mg (*p* = 0.001), and the GGT (*p* < 0.001) levels.

### 3.3. Blood Morphology

The basic blood parameters of the weaned offspring from the mothers that were exposed to FB during pregnancy are shown in Table 3.

No differences in the number of WBC or the percentage of monocytes were observed between the groups of female rat offspring. However, since both of the FB doses tended to increase the lymphocyte counts and decrease the granulocyte counts, linear effects were observed (*p* = 0.031 and *p* = 0.039, respectively). Maternal FB intoxication resulted in an increase in the RBC in the females, irrespective of the FB dose (linear and quadratic, *p* < 0.001 and *p* = 0.001, respectively). Significant linear increases in the HGB (*p* < 0.001) and the HCT (*p* < 0.001) were also observed following FB exposure, with the highest values being noted in the female offspring from the mothers that were intoxicated with 90 mg/kg b.w. FB. No other changes in the blood basal morphology of the female offspring were observed. 

A significantly lower number of WBC was observed in the male offspring from the mothers that were intoxicated with 60 mg/kg b.w. FB (linear and quadratic, *p* = 0.009 and *p* = 0.011, respectively). Maternal FB intoxication resulted in an increase in the RBC and the HGB, irrespective of the FB dose (linear and quadratic for both, *p* < 0.001 and *p* < 0.001, and *p* < 0.001 and *p* = 0.091, respectively). Moreover, a significant linear increase in the HCT was noted, with the highest value being observed in the 90 mg/kg b.w. FB group (*p* = 0.003). The number of platelets in the 90 mg/kg b.w.-FB-intoxicated group was significantly lower than that noted in the group that was intoxicated with 60 mg/kg b.w. (quadratic, *p* = 0.011).

No significant interaction effects were observed in the blood morphology parameters that were evaluated.

### 3.4. Liver Tissue Analysis

The liver structural parameters and the cell death parameters are presented in Table 4.

**Figure 1:**
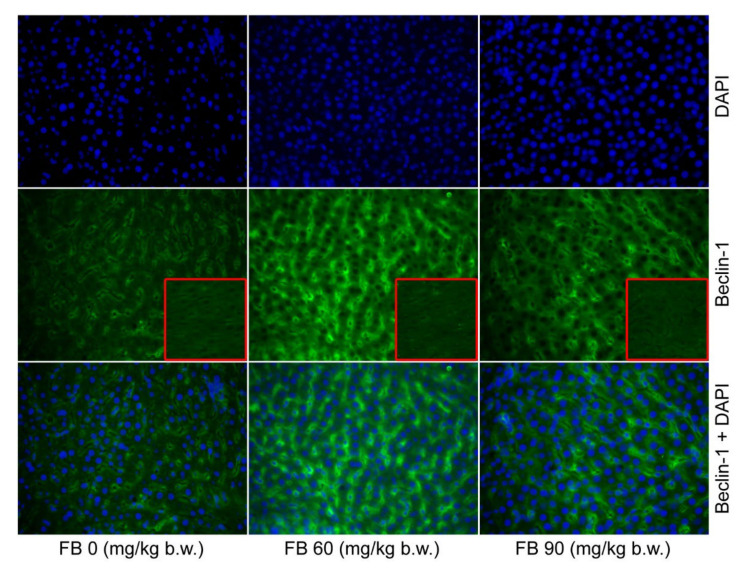
Effect of maternal FB intoxication on the level of autophagy (detected by Beclin-1 level) in the livers of offspring upon weaning, at 28 days of age. The red square inserts represent primary antibody controls. Magnification ×200.

**Figure 2:**
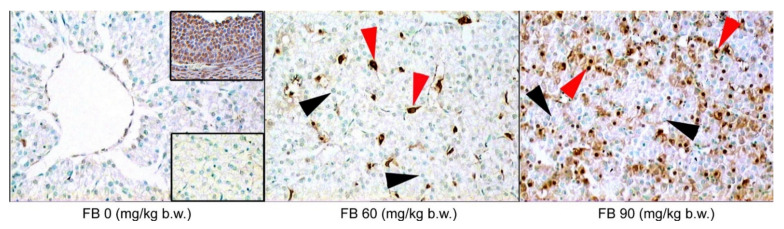
Effect of maternal FB intoxication apoptosis in liver (detected by the TUNEL reaction) of female offspring upon weaning, at 28 days of age. The black arrowheads indicate normal hepatocyte nuclei, and the red arrowheads indicate apoptotic cells. Upper insert–positive control of the TUNEL reaction–apoptotic granulosa cells in a rat preovulatory follicle. Lower insert–negative control of the TUNEL reaction–liver. Magnification ×200. Additional photos are included in the Supplementary Materials in Figure S1.

A significantly higher number of mononucleated hepatocytes were observed in the livers of the female rats in the 60 mg/kg b.w. FB group compared to that of the 0 FB group (quadratic, *p* = 0.017). The number of other cells (mainly leucocytes and Kuppfer cells) significantly increased after the prenatal FB exposure, with the highest number of other cells being observed in the group of the female rats that were exposed to FB at a dose of 60 mg/kg b.w. (linear and quadratic, *p* = 0.005 and *p* < 0.001). This parameter was also strongly gender-dependent (*p* < 0.001). Beclin-1, which is an autophagy protein, was predominant in both of the groups that were prenatally exposed to FB, with the higher number of Beclin-1 positive cells being observed in the 60 mg/kg b.w. FB group (linear and quadratic, *p* < 0.001 and *p* < 0.001) (Table 4, Figure 1). The number of apoptotic cells also increased after the FB exposure, with the highest number of apoptotic cells being observed in the 90 mg/kg b.w. FB group (linear and quadratic, *p* < 0.001 and *p* < 0.001) (Table 4, Figure 2). No other changes were observed in the livers of the female rats.

The number of binucleated hepatocytes in the livers of the male rats was significantly higher in the 90 mg/kg b.w. FB group compared to the others (linear, *p* = 0.004). The number of other cells was significantly decreased in the male rats after the prenatal FB exposure (linear and quadratic, *p* < 0.001 and *p* < 0.001). The number of cells showing autophagy (Beclin-1 positive) increased after the FB exposure, and this effect was linear and quadratic (*p* < 0.001) (Table 4, Figure 1). The number of apoptotic cells also significantly increased following the FB exposure, with the highest number of apoptotic cells being observed in the group that was exposed to FB at a dose of 90 mg/kg b.w. (linear and quadratic, *p* < 0.001 and *p* < 0.001) (Table 4, Figure 2).

### 3.5. Histopathological Tissue Assessment

**Figure 3:**
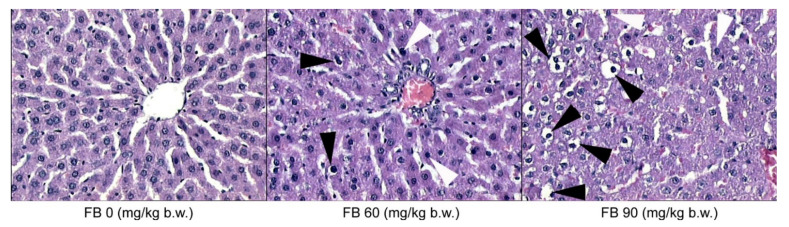
Effect of maternal FB intoxication on the liver structure of male offspring upon weaning, at 28 days of age. The black arrowheads indicate ballooning and the white arrowheads indicate degenerative chromatin condensation. Magnification ×200.

The microscopic evaluation of the histological structure of the liver showed no significant differences in the distribution of the portal triad and the terminal hepatic veins in the FB-exposed offspring compared to the unexposed offspring. The prenatal FB exposure had no effect on the spatial distribution of the lobules in the liver tissue, which was observed at a low magnification. However, only the livers of the control group had normal hepatocyte architecture, where all of the cells that were listed were of a similar size and hexagonal shape, with a centrally located nucleus and a homogeneous cytoplasm. The first signs of abnormalities in the structure and the spatial arrangement of the liver cells were observed in the offspring from the mothers that were exposed to the low dose of FB. The hepatic abnormalities were dramatically increased in the offspring from the mothers that were exposed to the high dose of FB, where the irregular shape and distribution of the hepatocytes were evident, irrespective of the offspring sex. Large abnormalities of the parenchymal structure in the liver lobules were also observed after maternal exposure to the high FB dose (Figure 3). Degenerative changes in the liver parenchyma, such as degenerative chromatin condensation that was directed toward apoptosis and ballooning, were observed in the offspring of both sexes that were prenatally exposed to the low dose of FB. These degenerative changes gradually increased in number and intensity in the livers of all of the offspring that were prenatally exposed to the high FB dose. Although no fibrotic changes were observed following the prenatal exposure to FB, vacuolization was observed in the livers of both of the FB-intoxicated groups, irrespective of sex, which is usually associated with decreased tissue oxygenation. Abnormalities in the course and the formation of the hepatic sinuses in both sexes were also observed after maternal intoxication with the high dose of FB (Figure 3).

**Figure 4:**
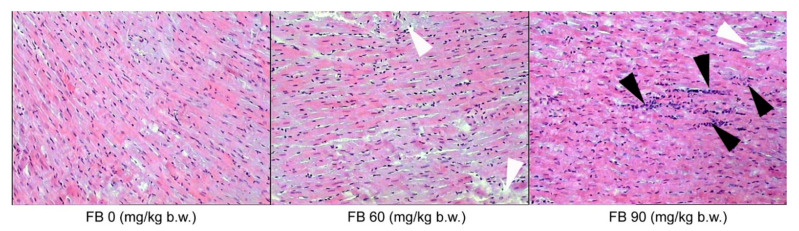
Effect of maternal FB intoxication on the heart structure of female offspring upon weaning, at 28 days of age. The lack arrowheads indicate inflammatory cell infiltration in the myocardium and the white arrowheads indicate tissue weakness and probable ischemic injury. Magnification ×200.

The histological sections of the left ventricular myocardium showed varying degrees of inflammatory cell infiltration in the myocardium of the rats of both sexes. No inflammation was observed in the control group, with very little inflammation being observed in the offspring from the low-dose FB group. However, moderate inflammatory cell infiltration was observed in the myocardium of the offspring that were exposed to the high dose of FB, irrespective of sex. Ischemic damage was also observed after maternal intoxication with both the low and the high dose of FB in the rats of both sexes (Figure 4).

**Figure 5:**
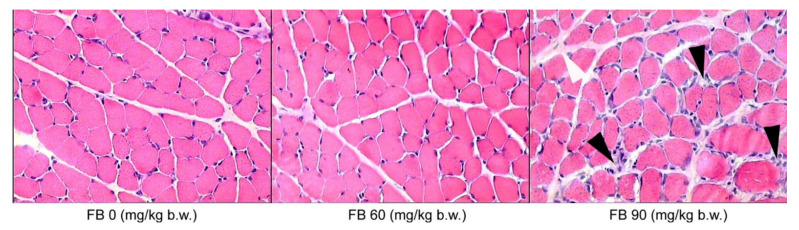
Effect of maternal FB intoxication on the structure of the biceps brachii muscles of male offspring upon weaning, at 28 days of age. The black arrowheads indicate connective tissue cell expansion and the white arrowhead indicates connective tissue matrix overgrowth. Magnification ×200.

In the case of the biceps brachii muscles, inflammatory cell infiltration was only observed in the groups that were prenatally exposed to FB, irrespective of the sex of the rats, where there was an expansion of connective tissue cells and hypertrophy of the connective tissue matrix (Figure 5).

## 4. Discussion

When considering the effect of FB on lipid metabolism, one should begin by looking at the role that ceramide plays in signal transduction and its’ biological effects. Fumonisin-induced toxicity is linked with the disturbance of sphingolipid synthesis. FB1 blocks the biosynthesis of de novo sphingolipids through the inhibition of ceramide synthases, which are the enzymes that participate in the creation of the ceramides. Sphingolipids are lipids, not static structural components of each cell’s bilayer membrane, that function as lipid second messengers. There are both simple and complex sphingolipids. The group of complex sphingolipids includes cerebrosides, gangliosides, and sphingomyelins. Sphingomyelins are hydrolyzed by sphingomyelinases into phosphocholine and ceramides. The ceramides are bioactive lipids that are generated in response to extracellular stimulation (by TNFα or IL-1 and IL-6) in numerous cell types, such as mammalian cells of hematopoietic or neural origin [25]. They regulate many physiological and pathological cellular processes, such as cell proliferation, differentiation, apoptosis, programmed cell death, inflammation, and even neuronal death during development [26]. The ceramides can be de novo synthesized from serine and palmitoyl CoA in the endoplasmic reticulum in the presence of ceramide synthases (CerS; e.g., sphingosine N-acyltransferase) [27]. Six mammalian CerS (CerS1–CerS6) are integral membrane proteins of the endoplasmic reticulum; they are found in various tissues [28]. CerS1, CerS5, and CerS6 are particularly implicated in cell signaling. CerS2, CerS4, and CerS6 are highly expressed during cancerous processes. The degradation of CerS2 results in long-term apoptosis and proliferation, which promotes carcinogenesis. The downregulation of CerS6 results in stress of the endoplasmic reticulum, leading to apoptosis, while CerS2 functions in autophagy, but not in apoptosis. In addition to the marked hepatopathy, the lack of CerS1 and CerS2 is linked with cerebellar ataxia and multiple structural changes in the peripheral and central nervous systems, respectively. CerS1 is highly expressed in Purkinje cells, while there is an abundance of CerS2 and CerS5 in the liver. CerS3 is highly expressed in the testes and it participates in male fertility; while CerS6 is found in the mammary glands and in the lungs [29].

The ceramides induce apoptosis, regulate cell differentiation, immunity, and participate in inflammatory responses. They are well-known antiproliferative agents and mediators of programmed cell death [30]. It has been reported that the ceramides activate various inflammatory signaling pathways, including the JAK2/STAT3 and the NF-κB pathways. Ceramide 6 has been linked with the phosphorylation of JAK2, which in turn initiates STAT3, which plays a key role in cell migration, reparation, and immune response dysregulation [26]. This pathway involves a group of proteins that act as negative-feedback inhibitors of the signaling that is induced by the cytokines that act via the JAK/STAT pathway [31]. These proteins are known as the suppressors of cytokine signaling proteins (SOCS: CIS, SOCS1–7) and they are implicated in the regulation of IL-6, leptin, granulocyte-colony-stimulating factor, IL-10, and growth hormone. SOCS1 is a JAK-binding protein, while SOCS3 is essential for both IL-6 and leptin signaling [32]. The ceramides also regulate the transcription factor NF-κB and they act on phospholipase A2 and cyclo-oxygenase, which are important for the synergistic induction of the prostaglandins in response to the ceramides [33]. The ceramides can modulate cell death by the inhibition of the Pi3K anti-apoptotic/pro-survival pathway through the modulation of Akt activity [34].

The ceramides are released through the production of free sphingosine, which in turn is metabolized into ceramides by CerS in another cellular compartment. This salvage pathway is involved in PKC signaling. The most active CerS in the salvage-derived ceramide pathway is CerS5, which enhances the phosphorylation of the p38-MAPK member of MAPKs (serine/threonine kinases). CerS5, through the PKC/p38-MAPK pathway, plays a role in stress-induced cell death [29]. MAPKs activation results in the phosphorylation and then the activation of the cytoplasmic and nuclear target proteins that are involved in proliferation, differentiation, migration, and survival. The ceramide-induced MAPK pathway activation regulates the mitochondrial changes. The P38-MAPK activity is crucial for proper immune and inflammatory responses. The ceramide-dependent p38-MAPK-mediated apoptosis is important in tissue homeostasis [35]. The signaling pathways involving the ceramides are presented in Figure S2 and some of the biological effects that are exerted by these pathways are presented in Figure S3 in the Supplementary Materials. 

As has been mentioned above, all FB inhibit ceramide synthetase. Furthermore, FB2 inhibits the protein serine/threonine phosphatases. Moreover, the FB-induced CerS inhibition reduces the activation of caspase 3 and 7 and prevents the cellular membrane permeabilization. FB1 is a causative factor of a near-complete reduction in the ceramide levels [29].

**Figure 6:**
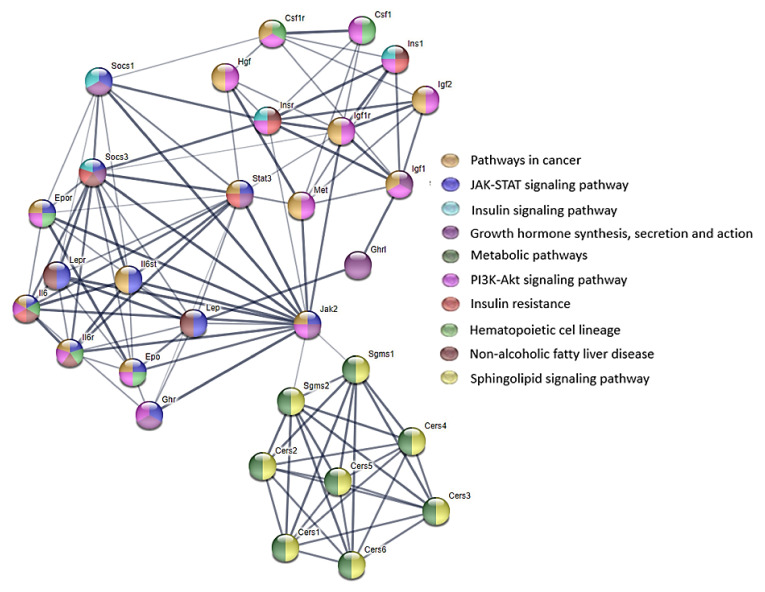
STRING protein–protein interaction diagram. STRING protein–protein network connectivity in signaling pathways involving lipid-biosynthesis-related proteins inhibited by FB (ceramide synthase and serine/threonine phosphatase) in a rat model. The thickest edge indicates the highest confidence in protein–protein association. The network contains 30 nodes. The analysis of pathways revealed that the presented proteins are involved in many biological signaling transduction pathways; https://string-db.org/, accessed on 24 July 2022.

**Figure 7:**
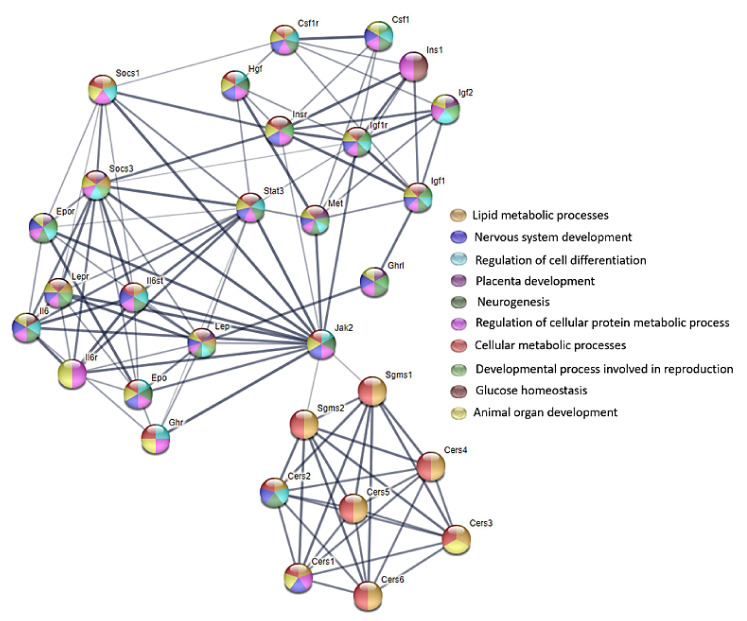
STRING protein–protein interaction diagram in various biological processes involving lipid-biosynthesis-related proteins inhibited by FB (ceramide synthase and serine/threonine phosphatase) in a rat model; https://string-db.org/, accessed on 17 June 2022. The thickest edge indicates the highest confidence in protein–protein association. The network contains 30 nodes. Gene ontology pathway analysis revealed that the presented proteins are involved in many biological pathways; https://string-db.org/, accessed on 24 July 2022.

Nutrition during the prenatal period plays an important role in the structural and functional development of mammals and it has long-term effects that are evident later on in life. The exposure to FB via the diet during these developmentally plastic periods has detrimental effects that are evident later on in life, especially since FB are able to cross the placental barrier [22]. The FB doses (60 and 90 mg/kg b.w.) that were used in the current study, although they seem to be high, did not result in any fatalities in either the dams or the offspring. Contrary to the results of the present study, Collins et al. [36,37] observed fetal toxicity with an increased number of late deaths, or the resorption of entire litters, after the oral administration of FB at a dose of 50 mg/kg b.w.; however, they used purified FB1 and mycotoxin, which were given during the early developmental period, starting from the 3rd day of gestation. This is probably the primary reason why the same effects were not observed in the current study, in which the maternal exposure to FB started from the 6th day of gestation. However, in a previous study that focused on postnatal FB intoxication, hepatotoxic effects, as well as inhibitory effects with regards to the body mass of adolescent rats, were observed [12]. Another study that focused on the dose-dependent effects of FB toxicity on the fetal development, showed not only hepatotoxic effects, but also detrimental sex-dependent effects in relation to the bone development of the newborns [19,20]. Although, Collins et al. [36] have presented both dose-dependent and sex-dependent effects of FB1 on the fetal body weight, their studies have also shown that FB1 that is given to pregnant dams does not affect the organ weights of either the fetal rats and the adult rats. The developmental effects of FB exposure have been investigated by Ferguson et al. [37], who used FB1 at doses that were in the range of 0.8–9.6 mg/kg b.w., during gestational days of 13–20, in rats. Their results have shown that FB1, irrespective of the dose, causes minimal developmental changes in a sex-specific manner. Another previous study showed that FB1 that is given at a dose of 60 mg/kg b.w. decreased the relative litter weight and inhibited the rats’ bone development [38]. Moreover, the differences that have been observed in the influence of FB on various organs can result from the difference in the tissue location of the six mammalian CerS (CerS1–CerS6) (as mentioned above) [28]. For this reason, various biological effects that are exerted by the signaling pathways involving the ceramides can be observed (Figure 6 and Figure 7).

The results of the current study agree with those of the previous studies showing a general inhibitory effect; however, our weaned rats weighed significantly less at birth than the control rats, regardless of the FB dose or the sex. Furthermore, at weaning, an increase in body mass was observed, and the neonatal growth of the offspring was not proportional, which is indicated by the absolute and relative weights of many of the vital organs, including the gonads, where many of the FB-induced effects were dose-dependent, irrespective of the sex of the offspring. The disturbances in the neonatal growth were linked with a decreased the GH concentration following the maternal FB exposure. Moreover, the final body weight and the disproportional organ development, as well as the serum insulin and the glucose concentrations, could indicate obesity and the risk of diabetes mellitus, which can occur particularly in female offspring, where at a similar insulin concentration to the male offspring, they had a higher glucose concentration. These findings could be in agreement with the Developmental Origin of Health and Disease (DOHaD) theory, which speaks of disturbances in the intrauterine environment that can increase the risk of the development of many diseases (such as diabetes and obesity) later on in life [39]. This risk can be strengthened by the phenomenon known as catch-up growth, which is accelerated growth that occurs between birth and the first few months of neonatal life. In the current study, the prenatal FB exposure led to electrolyte disturbances, which were not only dose-dependent but also sex-dependent. The changes in both the Ca and the P concentrations could be linked with impaired bone growth and development. Furthermore, the changes in the serum Mg concentrations could result in neurological complications, because Mg is necessary for the maintenance of the electric potential of the neurons [40]. It is also necessary for normal muscle function and is involved in many physiological processes as a cofactor in more than 300 enzymatic reactions that regulate the cellular processes. Magnesium also plays an important role in carbohydrate metabolism, glucose homeostasis, and insulin action, including both of the insulin receptor responses. The regulation of cellular glucose metabolism and insulin secretion by magnesium may be influenced by the interaction of magnesium with cellular calcium [41]. Mg is also involved in other metabolic processes, such as lipid metabolism and protein synthesis, and it is needed for the regulation of bone homeostasis [42]. It should be noted that the current study showed changes in the serum insulin, the glucose, and the Ca in the offspring that were prenatally exposed to FB, which were both dose-dependent and sex-dependent. Moreover, Ca^2+^ and Mg^2+^ ions are needed for the proper functioning of the nerves, and any changes in the serum Ca and Mg concentrations can result in functional impairments in the peripheral nervous system. FB1 is a natural ceramide synthetase inhibitor, and FB intoxication results in a reduction in the conduction velocity in the nerves and altered reflexes in rats [43]. Thus, in the case of any FB-induced electrolyte changes, it should be assumed that the neurons of peripheral nervous system is also affected. It has been proven that maternal exposure to FB results in FB-induced changes in certain neuronal populations of neurons of the enteric nervous system [21].

We also observed changes in the serum concentration of the total protein, which cannot be considered without taking into account the blood basal morphology, where the erythrogram showed an increase in the number of the RBC and other parameters that are related to the RBC. Given all of these changes, it is reasonable to assume that the offspring of the rats were most likely dehydrated after the FB exposure, or they suffered from renal impairment by FB (although the Na concentration and the renal histology were not assessed) or respiratory failure with asphyxia. A decrease in the lungs weight was observed following the FB exposure in the males. Future studies should perform a histological evaluation of the lungs tissue, especially since FB triggers pulmonary edema [44]. Moreover, the current study showed changes in the striated muscles, such as the heart and skeletal muscle. The CK (which is indicative of striated muscle injury) and the LDH (which is indicative of tissue damage, including the striated muscle and the liver) were increased in our weaned rats. This could indicate that ATP is more rapidly consumed by the muscles, and muscle failure (or acute kidney injury), including heart failure, occurred. Moreover, the current study also showed a decrease in the WBC in the males, which is directly indicative of intoxication, and was most likely caused by the infiltration of the muscle by leucocytes (Figure 4 and Figure 5). It can be assumed that these changes can lead to the weakening of the heart and skeletal muscles, and because the birth process is dependent on the functioning of the intercostal muscles, it can result in respiratory failure and an increase in some of the red blood cell parameters. The results of the current study are supported by an earlier study, in which the basal blood morphology was performed and an increase in both the HCT and the HCB was observed in broilers that were fed feed that was naturally contaminated with FB [45].

Liver injury was observed in the weaned offspring in the current study following the maternal FB intoxication, which was supported by the analysis of the ALAT, the ASPT, the ALP, the GGT, and the histopathological examination, including TUNEL reaction that was performed in order to determine apoptosis in the liver (Figure 4). It has been proven that FB contamination and consumption leads to cellular apoptosis [44] and FB is considered to be a non-genotoxic mycotoxin that triggers liver cancer in rats and other animals [46]. Moreover, the toxic effects of FB are linked with increased cytokine production, such as tumor necrosis factor and the interleukins (IL-1beta and IL-6), which cause inflammation in the gastrointestinal tract and are involved in the process of apoptosis [44]. Our current study also showed an increase in immunoreactive cells to the autophagy protein Beclin-1 in all of the FB-exposed groups, irrespective of the sex of the rats. 

## 5. Conclusions

The current study is the only study to our knowledge that has reported on the influence of prenatal FB exposure on the striated muscles, such as the heart and skeletal muscle, in which inflammation was observed. Moreover, the FB exposure resulted in the disproportional development of the rat offspring in a dose-dependent manner, which was probably caused by the bodily hormonal dysregulation. 

## Figures and Tables

**Table 1:** Body weight and relative organ weights of rat offspring following maternal exposure to 0, 60, or 90 mg/kg b.w. fumonisins.

Dependent Variable	Sex	FB (mg/kg b.w.)			*p*-Value	*p*-Level	
		0	60	90		Linear	Quadratic
Body weight at birth, g	F	8.10 ± 0.30 ^a^	6.40 ± 0.20 ^b^	5.90 ± 0.30 ^b^	<0.001	<0.001	0.071
End body weight, g		52.10 ± 2.10 ^c^	70.00 ± 1.40 ^a^	64.60 ± 1.40 ^b^	<0.001	<0.001	<0.001
Heart relative weight, %		0.56 ± 0.01 ^a^	0.47 ± 0.02 ^b^	0.48 ± 0.01 ^b^	<0.001	<0.001	0.012
Liver relative weight, %		4.50 ± 0.16 ^a^	4.35 ± 0.07 ^ab^	3.91 ± 0.10 ^b^	0.002	0.001	0.280
Kidney relative weight, %		1.18 ± 0.04	1.09 ± 0.04	1.06 ± 0.02	0.052	0.022	0.425
Lungs relative weight, %		1.30 ± 0.09	1.16 ± 0.09	1.10 ± 0.08	0.268	0.118	0.687
Ovaries relative weight, %		0.24 ± 0.02 ^b^	0.32 ± 0.02 ^a^	0.24 ± 0.02 ^b^	0.027	0.854	0.008
Body weight at birth, g	M	10.20 ± 0.30 ^a^	6.80 ± 0.10 ^b^	6.80 ± 0.20 ^b^	<0.001	<0.001	<0.001
End body weight, g		62.90 ± 1.70 ^c^	76.50 ± 1.90 ^a^	69.20 ± 0.80 ^b^	<0.001	0.007	<0.001
Heart relative weight, %		0.49 ± 0.02	0.47 ± 0.02	0.49 ± 0.02	0.712	0.636	0.456
Liver relative weight, %		4.43 ± 0.13 ^ab^	4.65 ± 0.12 ^a^	4.15 ± 0.04 ^b^	0.006	0.058	0.008
Kidney relative weight, %		1.14 ± 0.03	1.10 ± 0.04	1.08 ± 0.02	0.453	0.219	0.818
Lungs relative weight, %		1.43 ± 0.08 ^a^	1.04 ± 0.08 ^b^	1.14 ± 0.10 ^b^	0.007	0.018	0.024
Testis relative weight, %		1.15 ± 0.04 ^b^	1.26 ± 0.04 ^a^	1.16 ± 0.04 ^b^	0.131	0.889	0.046

Results are presented as mean ± SE with corresponding statistical analysis. FB—fumonisin, F—female, and M—male. Statistically significant differences between the groups (at *p*-value < 0.05) are indicated by ^a^, ^b^, and ^c^.

**Table 2:** Basal biochemical parameters of rat offspring following maternal exposure to 0, 60, or 90 mg/kg b.w. of fumonisins.

Dependent Variable	Sex	FB (mg/kg b.w.)			*p*-Value	*p*-Level	
		0	60	90		Linear	Quadratic
Insulin, ng/mL	F	0.17 ± 0.00 ^b^	0.18 ± 0.00 ^b^	0.30 ± 0.01 ^a^	<0.001	<0.001	<0.001
IL-1, pg/mL		16.59 ± 0.12 ^b^	19.37 ± 0.87 ^ab^	20.38 ± 1.42 ^a^	0.025	0.009	0.463
IL-6, pg/mL		15.57 ± 0.32 ^ab^	16.37 ± 0.59 ^a^	14.86 ± 0.07 ^b^	0.034	0.210	0.021
GH, ng/mL		9.24 ± 0,27 ^a^	7.45 ± 0,43 ^b^	5.92 ± 0,14 ^c^	<0.001	<0.001	0.735
Glucose, mmol/L		5.40 ± 0.17 ^b^	7.56 ± 0.15 ^a^	5.95 ± 0.09 ^b^	<0.001	0.007	<0.001
Total protein, g/L		35.79 ± 5.80 ^b^	60.64 ± 1.50 ^a^	57.49 ± 1.13 ^a^	<0.001	<0.001	0.003
Chol, mmol/L		0.95 ± 0.04	1.02 ± 0.05	0.98 ± 0.04	0.570	0.632	0.347
TBIL, µmol/L		1.60 ± 0.14	1.39 ± 0.15	1.58 ± 0.10	0.470	0.918	0.224
ASPT, U/L		297.66 ± 15.54 ^b^	410.17 ± 29.80 ^a^	466.67 ± 38.14 ^a^	<0.001	<0.001	0.442
ALAT, U/L		53.19 ± 1.45 ^b^	77.34 ± 4.48 ^a^	80.50 ± 4.50 ^a^	<0.001	<0.001	0.029
ALP, U/L		780.49 ± 45.21 ^a^	566.17 ± 60.61 ^b^	530.92 ± 22.40 ^b^	<0.001	<0.001	0.118
LDH, U/L		580.09 ± 24.67 ^b^	694.32 ± 28.36 ^a^	657.83 ± 36.89 ^a^	0.036	0.080	0.051
CK, U/L		225.32 ± 11.27 ^c^	917.87 ± 90.73 ^a^	562.92 ± 31.29 ^b^	<0.001	<0.001	<0.001
GGT, IU/L		0.25 ± 0.00 ^b^	0.35 ± 0.02 ^a^	0.27 ± 0.00 ^b^	<0.001	0.219	<0.001
Ca, mmol/L		2.83 ± 0.09	2.67 ± 0.01	2.75 ± 0.01	0.124	0.322	0.073
P, mmol/L		2.84 ± 0.03 ^a^	2.39 ± 0.09 ^b^	2.47 ± 0.11 ^b^	0.001	0.003	0.013
Mg, mmol/L		1.37 ± 0.16 ^a^	0.73 ± 0.05 ^b^	0.93 ± 0.03 ^b^	<0.001	0.003	0.001
Insulin, ng/mL	M	0.16 ± 0.00 ^b^	0.16 ± 0.01 ^b^	2.38 ± 0.07 ^a^	<0.001	<0.001	<0.001
IL-1, pg/mL		18.49 ± 0.06 ^ab^	20.54 ± 1.13 ^a^	17.33 ± 0.33 ^b^	0.008	0.236	0.003
IL-6, pg/mL		15.91 ± 0.37 ^a^	14.74 ± 0.02 ^b^	15.85 ± 0.41 ^ab^	0.025	0.900	0.007
GH, ng/mL		9.33 ± 0.06 ^a^	6.67 ± 0.05 ^b^	5.91 ± 0.05 ^b^	<0.001	<0.001	<0.001
Glucose, mmol/L		5.80 ± 0.27 ^b^	7.48 ± 0.38 ^a^	4.88 ± 0.11 ^c^	<0.001	0.025	<0.001
Total protein, g/L		55.89 ± 1.92	56.64 ± 2.20	58.15 ± 3.08	0.803	0.518	0.901
Chol, mmol/L		1.03 ± 0.15	1.02 ± 0.08	1.30 ± 0.17	0.271	0.167	0.396
TBIL, µmol/L		1.33 ± 0.17	1.55 ± 0.11	1.43 ± 0.08	0.465	0.574	0.273
ASPT, U/L		339.10 ± 9.12	365.92 ± 20.45	319.58 ± 9.477	0.080	0.311	0.043
ALAT, U/L		56.55 ± 3.01 ^b^	72.88 ± 1.75 ^a^	68.33 ± 2.12 ^a^	<0.001	0.001	<0.001
ALP, U/L		756.09 ± 18.07 ^ab^	823.41 ± 27.13 ^a^	614.25 ± 22.04 ^b^	<0.001	<0.001	<0.001
LDH, U/L		588.55 ± 6.42 ^b^	712.34 ± 21.78 ^a^	623.78 ± 23.42 ^b^	<0.001	0.195	<0.001
CK, U/L		236.62 ± 21.68 ^b^	703.10 ± 27.27 ^a^	591.50 ± 73.28 ^a^	<0.001	<0.001	<0.001
GGT, IU/L		0.38 ± 0.03 ^a^	0.28 ± 0.02 ^b^	0.42 ± 0.03 ^a^	0.001	0.265	<0.001
Ca, mmol/L		2.72 ± 0.05 ^b^	2.88 ± 0.01 ^a^	2.56 ± 0.06 ^c^	<0.001	0.013	<0.001
P, mmol/L		2.40 ± 0.05	2.34 ± 0.03	2.33 ± 0.04	0.404	0.232	0.541
Mg, mmol/L		0.99 ± 0.10	1.14 ± 0.12	0.95 ± 0.05	0.331	0.741	0.150

IL-1—interleukin 1 β; IL-6—interleukin 6; GH—growth hormone; Chol—cholesterol; TBIL—total bilirubin; ASPT—aspartate transaminase; ALAT—alanine transaminase; ALP—alkaline phosphatase; LDH—lactate dehydrogenase; CK—creatine kinase; GGT—gamma-glutamyl transferase; Ca—calcium; P—phosphorus; and Mg—magnesium. Results are presented as mean ± SE with corresponding statistical analysis. FB—fumonisin, F—female, and M—male. Statistically significant differences between the groups (at *p*-value < 0.05) are indicated by ^a^, ^b^, and ^c^.

**Table 3:** Basal blood parameters of rat offspring following maternal exposure to 0, 60, or 90 mg/kg b.w. of fumonisins.

Dependent Variable	Sex	FB (mg/kg b.w.)			*p*-Value	*p*-Level	
		0	60	90		Linear	Quadratic
WBC, 10^9^/L	F	7.73 ± 0.25	6.92 ± 0.21	6.83 ± 0.63	0.237	0.124	0.476
LYM, %		68.63 ± 2.42	70.90 ± 0.87	73.80 ± 1.14	0.093	0.031	0.874
MON, %		5.36 ± 0.13	5.10 ± 0.19	5.35 ± 0.26	0.588	0.966	0.307
GRA, %		24.91 ± 1.79	24.01 ± 0.93	20.90 ± 1.09	0.094	0.039	0.499
RBC, 10^12^/L		4.98 ± 0.05 ^b^	5.75 ± 0.07 ^a^	5.93 ± 0.08 ^a^	<0.001	<0.001	0.001
HGB, g/L		108.90 ± 4.29 ^b^	126.30 ± 1.89 ^a^	135.80 ± 2.53 ^a^	<0.001	<0.001	0.301
HCT, %		30.29 ± 1.00 ^b^	33.96 ± 0.44 ^ab^	38.35 ± 2.11 ^a^	<0.001	<0.001	0.834
MCV, fl		60.08 ± 2.00	59.14 ± 0.93	58.59 ± 3.62	0.910	0.669	0.948
MCH, pg		21.68 ± 0.79	22.01 ± 0.35	22.88 ± 0.18	0.247	0.107	0.674
MCHC, g/L		361.30 ± 3.27	372.60 ± 1.78	437.80 ± 49.68	0.144	0.069	0.450
PLT, 10^9^/L		1046.91 ± 62.54	1007.50 ± 30.23	964.56 ± 17.34	0.381	0.168	0.972
WBC, 10^9^/L	M	7.91 ± 0.07 ^a^	6.01 ± 0.33 ^b^	6.50 ± 0.53 ^ab^	0.002	0.009	0.011
LYM, %		68.63 ± 2.42	70.90 ± 0.87	73.80 ± 1.14	0.486	0.300	0.548
MON, %		5.27 ± 0.30	5.22 ± 0.16	5.96 ± 0.36	0.138	0.096	0.266
GRA, %		24.20 ± 1.25	24.41 ± 1.04	25.91 ± 1.46	0.583	0.343	0.681
RBC, 10^12^/L		4.87 ± 0.07 ^b^	5.74 ± 0.14 ^a^	5.58 ± 0.10 ^a^	<0.001	<0.001	<0.001
HGB, g/L		105.90 ± 4.56 ^b^	123.40 ± 1.69 ^a^	127.50 ± 2.38 ^a^	<0.001	<0.001	0.091
HCT, %		29.60 ± 0.91 ^b^	33.67 ± 0.40 ^ab^	36.10 ± 2.29 ^b^	0.011	0.003	0.644
MCV, fl		60.84 ± 1.67	58.87 ± 0.87	57.94 ± 3.25	0.632	0.352	0.848
MCH, pg		21.75 ± 0.87	21.59 ± 0.26	24.10 ± 1.20	0.090	0.066	0.219
MCHC, g/L		360.70 ± 2.80	324.50 ± 19.16	423.10 ± 48.39	0.079	0.152	0.077
PLT, 10^9^/L		966.15 ± 22.23 ^ab^	1071.00 ± 25.95 ^a^	940.08 ± 51.53 ^b^	0.034	0.609	0.011

WBC—white blood cells; LYM—lymphocytes; MON—monocytes; GRA—granulocyte; RBC—red blood cells; HGB—hemoglobin; HCT—hematocrit; MCV—mean corpuscular volume; MCH—mean corpuscular hemoglobin; MCHC—mean corpuscular hemoglobin concentration; and PLT—platelets. Results are presented as mean ± SE with corresponding statistical analysis. FB—fumonisin, F—female, and M—male. Statistically significant differences between the groups (at *p*-value < 0.05) are indicated by ^a^ and ^b^.

**Table 4:** Liver structural parameters and cell-death-related parameters of rat offspring following maternal exposure to 0, 60, or 90 mg/kg b.w. of fumonisins.

Dependent Variable	Sex	FB (mg/kg b.w.)			*p*-Value	*p*-Level	
		0	60	90		Linear	Quadratic
Mononucleated hepatocyte number,/mm^2^	F	2069.7 ± 81.3 ^b^	2661.4 ± 143.5 ^a^	2260.8 ± 201.4 ^ab^	0.040	0.383	0.017
Binucleated hepatocyte number,/mm^2^		75.3 ± 9.9	61.1 ± 13.3	101.4 ± 17.1	0.145	0.199	0.127
Other cells,/mm^2^		232.8 ± 17.7 ^c^	1265.0 ± 72.4 ^a^	471.4 ± 50.8 ^b^	<0.001	0.005	<0.001
Beclin-1 positive cells,/100 cells		7.6 ± 0.2 ^c^	17.7 ± 0.5 ^a^	10.6 ± 0.3 ^b^	<0.001	<0.001	<0.001
Number of apoptotic cells,/100 cells		1.1 ± 0.2 ^c^	6.2 ± 0.4 ^b^	19.7 ± 1.0 ^a^	<0.001	<0.001	<0.001
Mononucleated hepatocyte number,/mm^2^	M	1781.4 ± 74.0	1842.2 ± 91.0	1548.6 ± 140.0	0.150	0.139	0.190
Binucleated hepatocyte number,/mm^2^		35.6 ± 4.2 ^b^	38.6 ± 7.1 ^b^	72.2 ± 10.3 ^a^	0.007	0.004	0.122
Other cells,/mm^2^		699.7 ± 14.5 ^a^	213.9 ± 16.7 ^c^	397.5 ± 41.9 ^b^	<0.001	<0.001	<0.001
Beclin-1 positive cells,/100 cells		8.2 ± 0.2 ^c^	18.3 ± 0.5 ^a^	11.1 ± 0.3 ^b^	<0.001	<0.001	<0.001
Number of apoptotic cells,/100 cells		1.2 ± 0.2 ^c^	6.2 ± 0.4 ^b^	20.0 ± 1.1 ^a^	<0.001	<0.001	<0.001

Results are presented as mean ± SE with corresponding statistical analysis. FB—fumonisin, F—female, and M—male. Statistically significant differences between the groups (at *p*-value < 0.05) are indicated by ^a^, ^b^, and ^c^.

## Data Availability

The data presented in this study are available on request from the corresponding authors.

## Supplementary Materials

The following supporting information can be downloaded at: https://www.mdpi.com/article/10.3390/ani12182353/s1, The list protein abbreviations in STRING protein-protein interaction diagram in Figure 6 and Figure 7 and Supplementary Figures S1 and S2. with description from UniProt database of their basic functions, Figure S1: Additional photos of the effect of maternal FB intoxication on liver apoptosis (detected by the TUNEL reaction); Figure S2: STRING protein-protein interaction diagram in various signaling pathways involving lipid biosynthesis-related proteins inhibited by FB (ceramide synthase and serine/threonine phosphatase); Figure S3: STRING protein-protein interaction diagram in various biological processes involving lipid biosynthesis-related proteins inhibited by FB (ceramide synthase and serine/threonine phosphatase).
