# Supplementary material for: Basal Blood Morphology, Serum Biochemistry, and the Liver and Muscle Structure of Weaned Wistar Rats Prenatally Exposed to Fumonisins
Source: Animals (Basel). 2022 Sep 8;12(18):2353. doi: 10.3390/ani12182353 (PMC9495192; doi:10.3390/ani12182353)
Supplement: Supplementary file 1 [file animals-12-02353-s001.zip › animals-1856993-supplementary.pdf]

Basal blood morphology, serum biochemistry, and the liver and muscle structure of weaned Wistar rats, prenatally exposed to fumonisins

Ewa Tomaszewska, Halyna Rudyk , Dorota Wojtysiak, Janine Donaldson, Siemowit Muszyński, Marcin B. Arciszewski, Nataliia Lisova, Oksana Brezvyn, Iwona Puzio, Beata Abramowicz, Marta Pawłowska-Olszewska, Ihor Kotsyumbas and Piotr Dobrowolski

The list protein abbreviations in STRING protein-protein interaction diagram in Figures 6,7 and supplementary Figures S1 and S2. with description from UniProt database via STRING of their basic functions.

Cers1,2,3,4,5,6	Ceramide synthase 1,2,3,4,5,6; Inhibited by fumonisin B1, especially Cers5;
Csf1	Macrophage colony-stimulating factor 1; This cytokine plays an crucial role in the regulation of proliferation, survival and differentiation of hematopoietic precursor cells (monocytes and macrophages);
Csf1r	The receptor of macrophage colony-stimulating factor 1;
Degs2	Sphingolipid delta(4)-desaturase/C4-monooxygenase DES2; Bifunctional enzyme which acts as both a sphingolipid delta(4)-desaturase and a sphingolipid C4-monooxygenase;
Epo	Erythropoietin; This hormone regulates erythrocyte differentiation and proliferation;
Epor	Receptor for erythropoietin. Mediates erythropoietin-induced erythroblast proliferation and differentiation;
Ghr	The receptor of growth hormone receptor; It is important for the regulation of postnatal development;
Ghrl	Appetite-regulating hormone; It stimulates the secretion of gastric acid, and is involved in growth regulation;
Hgf	Hepatocyte growth factor; hepatotrophic factor;
Igf1	Insulin-like growth factor I;
Igf1r	The receptor of insulin-like growth factor 1 receptor;
Igf2	Insulin-like growth factor II; The main growth hormone during prenatal time in mammals. It regulates tissue differentiation and feto-placental development;
Il6	Interleukin-6;
Il6r	Interleukin receptor;
Ins1	Insulin-1;
Jak2	Tyrosine-protein kinase JAK2; In the cytoplasm, It plays a essential role in signal transmission through its link with receptors like prolactin (PRLR), growth hormone (GHR), erythropoietin (EPOR), leptin (LEPR), thrombopoietin (THPO);
Lep	Leptin; It regulates energy homeostasis and controls body weight;
Lepr	Receptor for hormone leptin;
Map3K11	Mitogen-activated protein kinase 11; It participates in the JUN N-terminal pathway.
Mapk12	Serine/threonine kinase, an essential component of the MAP kinase signal transduction pathway;
Met	Receptor tyrosine kinase which transduces signal from the extracellular matrix into the cell (the cytoplasm) by connection to hepatocyte growth factor/HGF ligand;
Sgms1,2	Sphingomyelin synthase 1 and 2; They synthesize the sphingolipid (sphingomyelin);
Smpd1,2	Sphingomyelin phosphodiesterase 1,2; Converts sphingomyelin to ceramide;
Socs1,3	SOCs family proteins. Socs1 and 3 are involved in the negative regulation through the JAK/STAT3 pathway. Socs3 can inhibit cytokine signal transduction through binding to the receptors of tyrosine kinase including insulin, erythropoietin , IL12, and leptin receptors. Through the connection to JAK2, it inhibits its kinase activity, and lead to the suppression of fetal liver erythropoiesis;
Stat3	Signal activator and transducer of transcription 3 that participate in the mediation of cellular responses to leptin, Ils, and other growth factors.

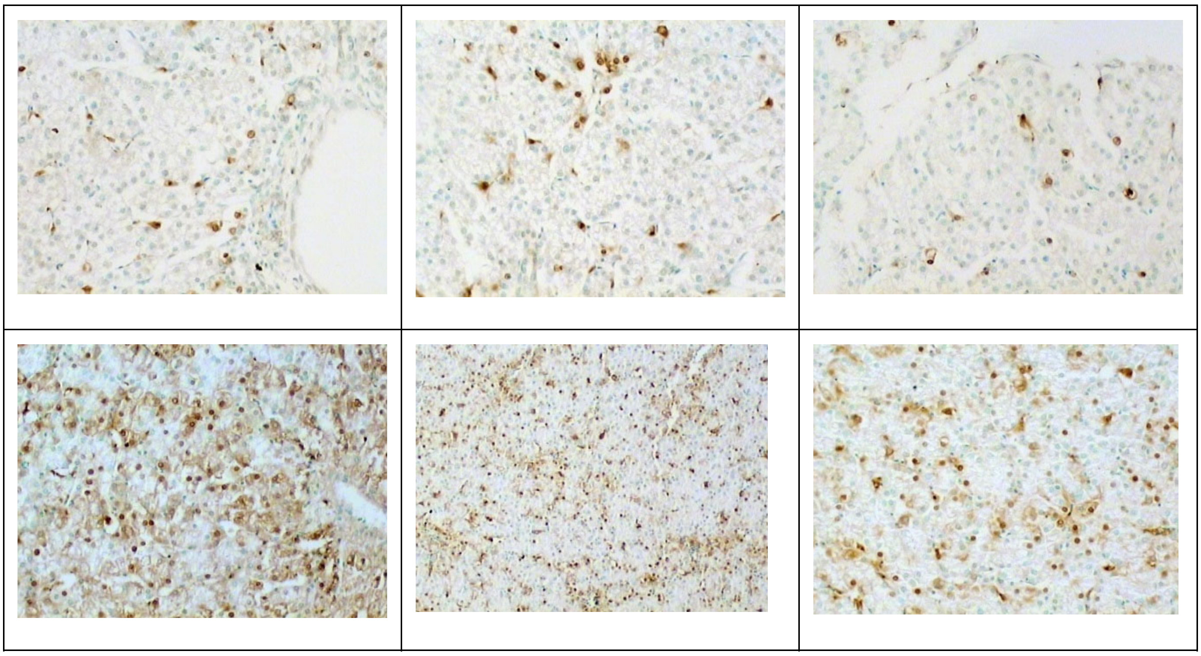

Figure S1. Additional photos of the effect of maternal FB intoxication on liver apoptosis (detected by the TUNEL reaction). Magnification x200.

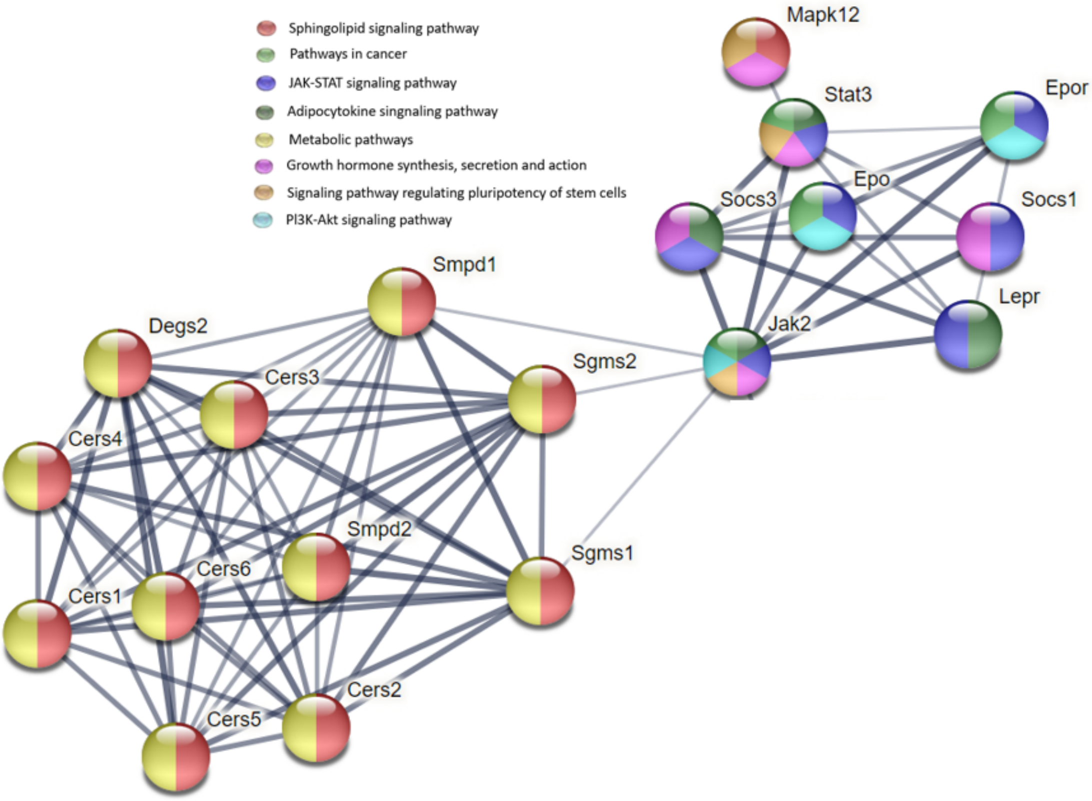

Figure S2. STRING protein-protein interaction diagram in various signaling pathways involving lipid biosynthesis-related proteins inhibited by FB (ceramide synthase and serine/threonine phosphatase). The thickest edge indicates the highest confidence in protein-protein association. Gene ontology pathway analysis revealed that the proteins presented here are involved in many biological pathways. Network contains 19 nodes; https://string-db.org/, accessed on 24.07.2022.

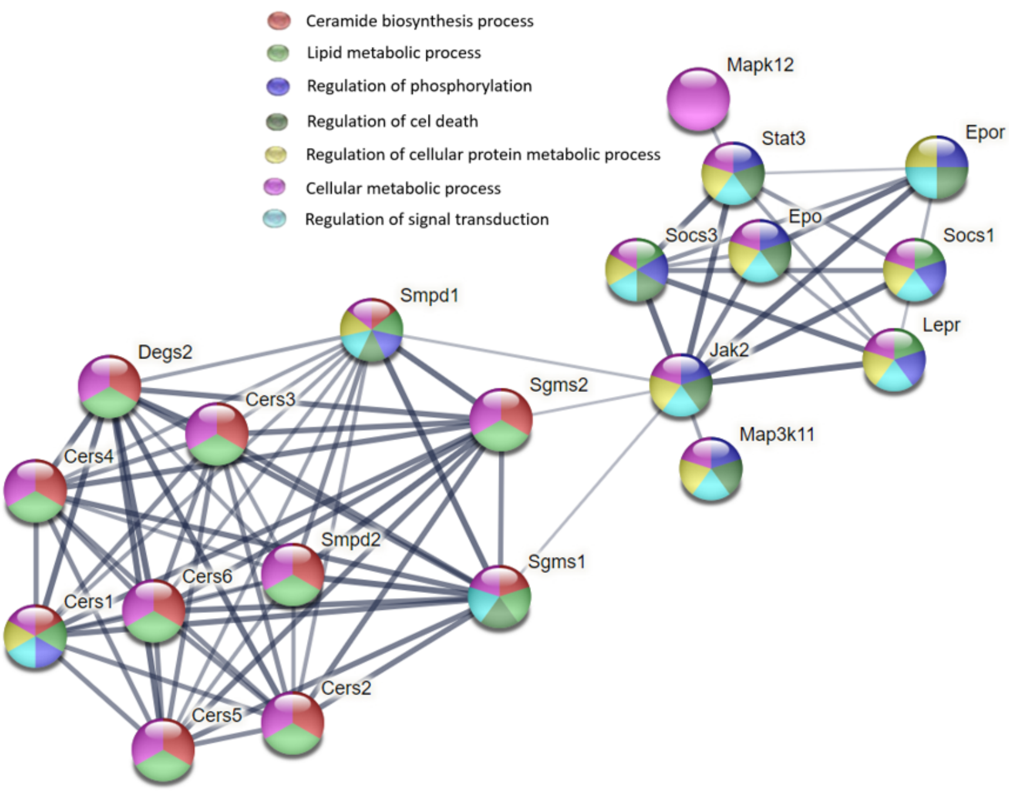

Figure S3. STRING protein-protein interaction diagram in various biological processes involving lipid biosynthesis-related proteins inhibited by FB (ceramide synthase and serine/threonine phosphatase). The thickest edge indicates the highest confidence in protein-protein association. Network contains 20 nodes. The analysis of biological effects revealed that the proteins presented here are involved in many biological functions within the organism; https://string-db.org/, accessed on 24.07.2022.
